# Distinct expression patterns of Hedgehog signaling components in mouse gustatory system during postnatal tongue development and adult homeostasis

**DOI:** 10.1371/journal.pone.0294835

**Published:** 2024-06-07

**Authors:** Archana Kumari, Nicole E. Franks, Libo Li, Gabrielle Audu, Sarah Liskowicz, John D. Johnson, Charlotte M. Mistretta, Benjamin L. Allen

**Affiliations:** 1 Department of Biologic and Materials Sciences & Prosthodontics, School of Dentistry, University of Michigan, Ann Arbor, Michigan, United States of America; 2 Department of Cell and Developmental Biology, Medical School, University of Michigan, Ann Arbor, Michigan, United States of America; 3 Department of Cell Biology and Neuroscience, Rowan-Virtua School of Translational Biomedical Engineering and Sciences, Virtua Health College of Medicine and Life Sciences of Rowan University, Stratford, New Jersey, United States of America; 4 Department of Biology, University of Scranton, Scranton, Pennsylvania, United States of America; 5 Rowan-Virtua School of Osteopathic Medicine, Virtua Health College of Medicine and Life Sciences of Rowan University, Stratford, New Jersey, United States of America; AERE: Atomic Energy Research Establishment, BANGLADESH

## Abstract

The Hedgehog (HH) pathway regulates embryonic development of anterior tongue taste fungiform papilla (FP) and the posterior circumvallate (CVP) and foliate (FOP) taste papillae. HH signaling also mediates taste organ maintenance and regeneration in adults. However, there are knowledge gaps in HH pathway component expression during postnatal taste organ differentiation and maturation. Importantly, the HH transcriptional effectors GLI1, GLI2 and GLI3 have not been investigated in early postnatal stages; the HH receptors PTCH1, GAS1, CDON and HHIP, required to either drive HH pathway activation or antagonism, also remain unexplored. Using *lacZ* reporter mouse models, we mapped expression of the HH ligand SHH, HH receptors, and GLI transcription factors in FP, CVP and FOP in early and late postnatal and adult stages. In adults we also studied the soft palate, and the geniculate and trigeminal ganglia, which extend afferent fibers to the anterior tongue. *Shh* and *Gas1* are the only components that were consistently expressed within taste buds of all three papillae and the soft palate. In the first postnatal week, we observed broad expression of HH signaling components in FP and adjacent, non-taste filiform (FILIF) papillae in epithelium or stroma and tongue muscles. Notably, we observed elimination of *Gli1* in FILIF and *Gas1* in muscles, and downregulation of *Ptch1* in lingual epithelium and of *Cdon*, *Gas1* and *Hhip* in stroma from late postnatal stages. Further, HH receptor expression patterns in CVP and FOP epithelium differed from anterior FP. Among all the components, only known positive regulators of HH signaling, SHH, *Ptch1*, *Gli1* and *Gli2*, were expressed in the ganglia. Our studies emphasize differential regulation of HH signaling in distinct postnatal developmental periods and in anterior versus posterior taste organs, and lay the foundation for functional studies to understand the roles of numerous HH signaling components in postnatal tongue development.

## Introduction

**Fig 1:**
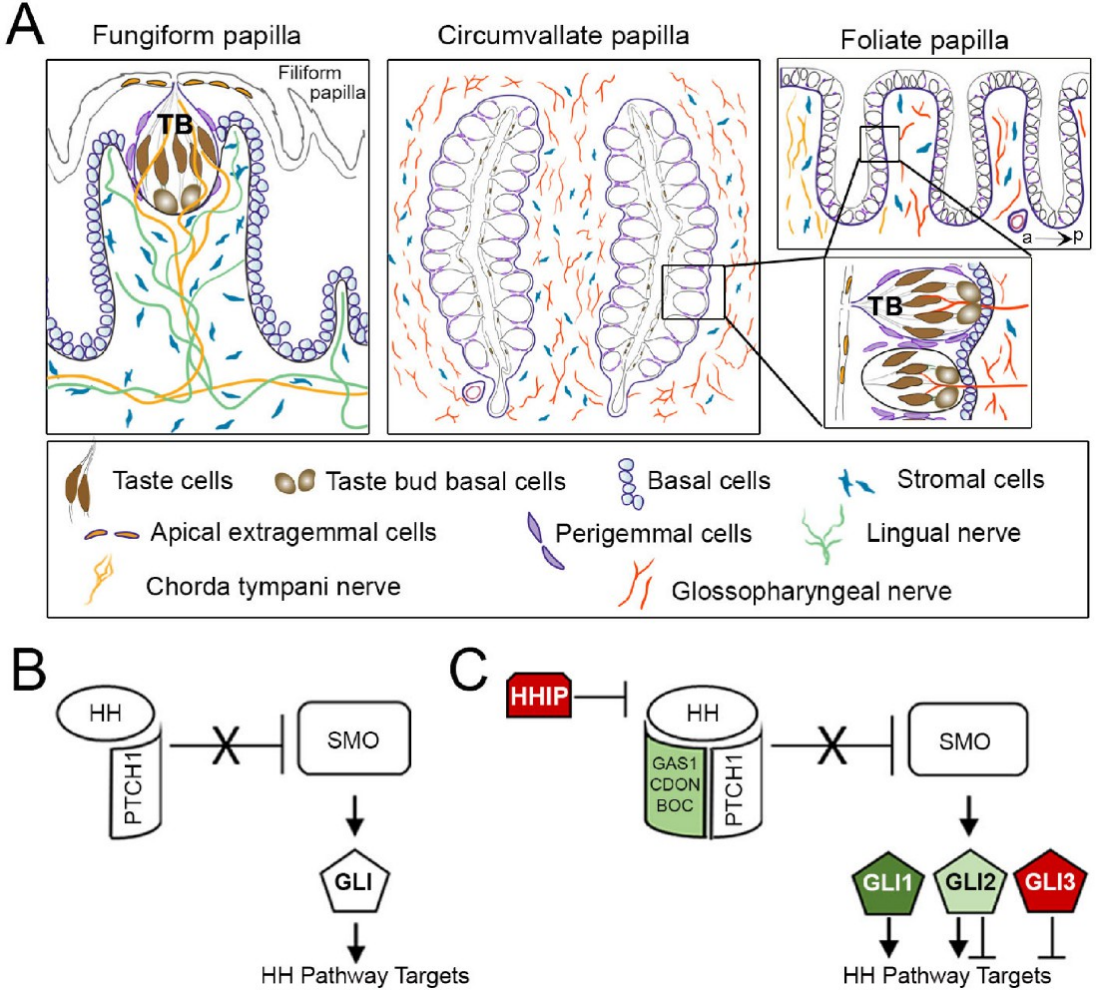
Schematic of lingual taste papillae and Hedgehog (HH) signaling. (A) Illustrations of tissue architecture for the fungiform (FP), circumvallate (CVP) and foliate (FOP) papillae. FP is presented in a sagittal section, adjacent to spinous, non-taste filiform papilla. The CVP and FOP are illustrated in a horizontal section (to the dorsal tongue surface), and boxes refer to magnified taste bud (TB) distribution along the epithelium. Each TB is composed of taste cells and TB basal cells, and is surrounded by perigemmal cells. All the papillae comprise basal cells in the epithelium, extragemmal cells on the apical surface and stromal cells in the connective tissue core. Gustatory (chorda tympani and glossopharyngeal) and mechanosensory (lingual) nerves are indicated. FOP anterior (a) and posterior (p) ridges are indicated. (B) HH ligand binds PTCH1, releasing SMO inhibition to modulate GLI proteins and activate HH target gene expression. (C) HH signaling is regulated by the co-receptors, GAS1, CDON and BOC (green, activator), and the secreted antagonist HHIP (red, repressor). Transcriptional readouts of HH signaling are controlled by three GLI proteins: GLI1 (green, activator), GLI2 (light green, activator>repressor), and GLI3 (red, repressor>activator).

The tongue houses taste buds in three different taste papillae at three different locations: fungiform papilla (FP) on the anterior two thirds, circumvallate papilla (CVP) on the posterior third mid-dorsum and foliate papilla (FOP) bilaterally on the posterior sides. These papilla types and resident taste buds develop with different time courses in mammalian species [1–3]. In sheep for example, the taste buds make a fetal appearance, continue development *in utero* and have extended postnatal maturation [4]. In rat and mouse, the lingual taste papillae emerge in the embryo; although initial taste buds-like cell clusters are seen before birth, taste buds *per se* do not emerge until postnatal stages [5–7]. However, both papillae and taste buds continue to develop during early postnatal stages and reach maturity in the anterior and posterior regions by the third and sixth postnatal weeks [1,8–10]. A mature FP harbors a single taste bud, while a CVP and multiple rows of FOP house numerous taste buds tightly associated with each other (**Fig 1A**). Anterior tongue and FP receive innervation from chorda tympani and lingual nerve fibers and posterior tongue and CVP are innervated by the glossopharyngeal nerve (**Fig 1A)**. Interestingly, FOP anterior ridges receive chorda tympani nerve fibers and posterior ridges are also innervated by the glossopharyngeal nerve (**Fig 1A)** [11–13]. The distinct location, morphology, and innervation of three lingual taste papillae may lead to their different trajectories of embryonic and postnatal development.

In our current study, we use mouse models. The number of mouse FP taste cells increases rapidly by postnatal day 7 (P7), more slowly between P7 and P14, and finally reaches a steady level by P21 [10,14,15]. In contrast, the number of rodent CVP and FOP taste buds increases threefold between P7 and P45 and reaches a maximum at P90 [1,10,16]. In addition, formation of taste pores, remodeling of gustatory innervation and enlargement of tongue muscles also occurs during the initial postnatal weeks [10,16–18]. These various and extreme changes must be tightly coordinated to create a stereotypical, functionally mature taste organ.

Hedgehog (HH) signaling is an essential regulator of embryonic patterning [19–21], postnatal organ development [22,23] and homeostasis [24] of several organs. We have previously shown the role of epithelial HH/GLI signaling in the maintenance of adult taste organs [25], and that HH signaling through Smoothened (SMO) also regulates papilla/taste buds homeostasis and neural responses [26–28]. HH signaling is also active and vital during tongue development [5,7,29,30]. However, few studies have investigated HH signaling regulation in the anterior tongue during the initial postnatal weeks when the dynamic papillae and taste buds are continuously growing [8,10,15]. Further, HH signaling regulation in posterior CVP and FOP remain to be investigated. Thus, identification of signaling component activities during postnatal lingual development and maturation is essential to understand HH regulatory roles.

Fundamentally, HH signaling initiates through ligand binding to the canonical receptor Patched 1 (PTCH1), which relieves inhibition of SMO, leading to downstream signaling through the modulation of GLI proteins and altered transcription of HH pathway targets (**Fig 1B**) [31–33]. Notably, HH pathway activation also requires ligand interactions with the co-receptors CDON, BOC and GAS1 [34], whereas the pathway is inhibited by binding of the HH antagonist HHIP to HH ligands (**Fig 1C**) [35,36]. Furthermore, HH transcription requires the combined use of three different GLI proteins. GLI1 is a transcriptional target and encodes an exclusive activator of the HH pathway; GLI2 is the major transcriptional activator; GLI3 acts principally as a repressor in HH signal transduction (**Fig 1C**) [37]. The field of taste research has mainly focused on the ligand, sonic HH (SHH), and the target gene, *Gli1*, while PTCH1, GLI2 and GLI3 have received less attention. Further, the obligatory membrane receptors GAS1, BOC and CDON and the HH antagonist HHIP remain largely unexplored. Our recent studies with HHIP [27] emphasize that these less studied HH pathway components might not be ‘secondary’ signaling elements but rather key signaling molecules that regulate organ integrity and thus need to be addressed.

As FP, CVP and FOP are distinct in their development and structural organization, their signaling regulation might be different. Thus, we investigated the expression pattern of HH signaling components in anterior FP and posterior CVP and FOP tissue regions: taste buds, basal, perigemmal, apical extragemmal and stromal cells (**Fig 1A**). We studied the ligand *Shh*, membrane receptors *Ptch1*, *Gas1*, *Cdon* and HH antagonist *Hhip*, and transcription factors *Gli1*, *Gli2* and *Gli3* in developing FP, CVP and FOP at the early postnatal stages between P3 and P10 (termed P7), and compared the pattern with that of mature taste papillae in mice aged eight weeks or older (termed adult). We also analyzed anterior FP during a later development stage between P21 and P31 (termed P28). In addition, we mapped HH pathway components in the adult soft palate, and geniculate and trigeminal ganglia with soma for chorda tympani and lingual nerve fibers, respectively, in the anterior tongue. We have used X-gal staining of *lacZ*- reporter mouse models or immunostaining to map the pathway components. The data reveal unique expression of the positive regulators of HH signaling activity, *Ptch1*, *Gas1*, *Cdon*, *Gli1* and *Gli2* in FP, CVP and FOP, and soft palate. Further differences are noted in the expression pattern of several HH signaling components in the anterior tongue, which is broader in the early postnatal stages as compared to adulthood. Notably, SHH, the receptor *Ptch1*, and the transcription factors *Gli1* and *Gli2* are the only components of the HH pathway observed in both geniculate and trigeminal ganglia. Overall, our data suggest a shift in HH pathway regulation of taste organs after the conclusion of postnatal tongue maturation.

## Materials and methods

### Mice

All animal use and care procedures were performed according to the guidelines of the National Institutes of Health. Informed written consent for all the protocols of the University of Michigan and Rowan University was obtained by the Institutional Animal Care and Use Committee (IACUC). Male or female mice, from the first postnatal week through adult stages were used. Pups less than 10 days of age were euthanized by decapitation, while in pups more than 10 days old, we used CO_2_ overdose, followed by decapitation.

### Mouse models/strains

#### *lacZ* reporters

Mice carrying *lacZ* alleles for the ligand (*Shh*^*lacZ/+*^, MGI:2678342) [38,39], HH-receptor *Ptch1* (*Ptch1*^*lacZ*/+^, Jackson Laboratories strain:003081) [40], HH co-receptor *Cdon* (*Cdon*^*lacZ/+*^) [41], HH antagonist *Hhip* (*Hhip*^*lacZ/+*^, Jackson Laboratories strain:006241) [42], HH target gene/responding *Gli1* (*Gli1*^*lacZ*/+^) (Jackson Laboratories strain:008211), HH transcriptional activator *Gli2* (*Gli2*^*lacZ/+*^, Jackson Laboratories strain:007922) [43] and transcriptional repressor *Gli3* (*Gli3*^*lacZ/+*^) [44] were maintained on a mixed 129S4/SvJaeJ/C57BL6/J background. HH co-receptor *Gas1* (*Gas1*^*lacZ/+*^) [20] was maintained on a C57BL6/J background.

#### RFP reporter

Mice carrying tamoxifen-inducible expression of RFP in *Shh*-expressing cells and their progeny (*Shh*^*CreERT2*^*;R26*^*RFP*^) were used. *Shh*^*CreERT2*^ (Jackson Laboratories strain:005623); *R26*^*RFP*^ (Jackson Laboratories strain:007914) double transgenic animals were given 400 mg/kg of tamoxifen in Teklad Global Diet (Harlan) daily for 14–30 days.

### Tissue dissection and processing

Tongues on mandibles were collected between postnatal day (P) 3 and P10 (termed P7), between P21 and P31 (termed P28) and between 8–15 weeks of age (termed adult). Soft palate (SP) and the ganglia (geniculate, GG and trigeminal, TG) were dissected at adult stages. All the tissues were fixed for 2h at 4°C in 4% paraformaldehyde in PBS. Fixed tongues were cut to obtain anterior two thirds to include fungiform (FP) and filiform (FILIF) papillae and the posterior third to include circumvallate (CV) and foliate (FOP) papillae. After fixation, all tissues were cryoprotected overnight with 30% sucrose in PBS and embedded in O.C.T. compound (Tissue-Tek, Sakura Finetek) for X-Gal staining or immunostaining as described previously [26,27]. Serial sagittal sections (FP), horizontal sections (CV, FOP, GG and TG) or coronal sections (SP) were cut at 10 μm for immunostaining [26,27].

### X-gal staining

Tissue sections were incubated in X-gal solution for 16-18h except for lingual *Gas1* and *Gli3* tissues which were incubated for 4h and 48h, respectively. X-gal staining was followed by immunostaining of taste bud cells (K8) or epithelium (Ecad) in FP sections and ligand (SHH) in GG and TG sections.

### Immunostaining

Immunoreactions were performed as described previously [26,27]. Briefly, tongue or ganglion sections were air dried, rehydrated, blocked (in 10% normal donkey serum, 0.3% Triton-X in PBS-X), and incubated overnight at 4°C with primary antibodies. On the next day, slides were washed and incubated with appropriate secondary antibodies for 1–2 h at room temperature in the dark. Primary antibodies were goat anti-SHH (AF464, 0.1 μg/mL; R&D Systems); rat anti-keratin 8 (TROMA-1, 1:1,000; Developmental Studies Hybridoma Bank); goat anti–E-cadherin (AF748, 1:5,000; R&D Systems) and rabbit anti-RFP (600-401-379, 1:1,000; Rockland). For SHH in tongue sections, the heat-induced antigen-retrieval method [26] was used.

### Imaging

Tissue section images were acquired with a Nikon Eclipse 80i microscope and Nikon DS Ri2 camera system or an automated Keyence BZ-X810 microscope. Photomicrographs were adjusted for brightness and contrast in parallel across one figure and assembled with Adobe Photoshop.

### Data analyses

We examined a minimum of 8 FP per tongue for each reporter line at each developmental stage. We also utilized 2–4 mouse tongues of the same postnatal age for analyzing FP, CVP and FOP, 2–3 SP, and GG and TG per line. X-gal expression was studied in serial sections, and the consistent presence of blue-colored product was considered as expression. We examined expression in the cell types depicted in the **Fig 1A** legend. The expression is categorized as ‘no’ if there is no blue product in that area, ‘partial’ if only few cells within the region are stained blue and ‘complete’ indicates that all cells are positive for X-gal staining. The expression patten was confirmed in replicates for all the different genes.

## Results

### Expression pattern of *Shh* ligand and membrane receptors *Ptch1*, *Gas1*, *Cdon* and *Hhip* in the anterior tongue

**Fig 2:**
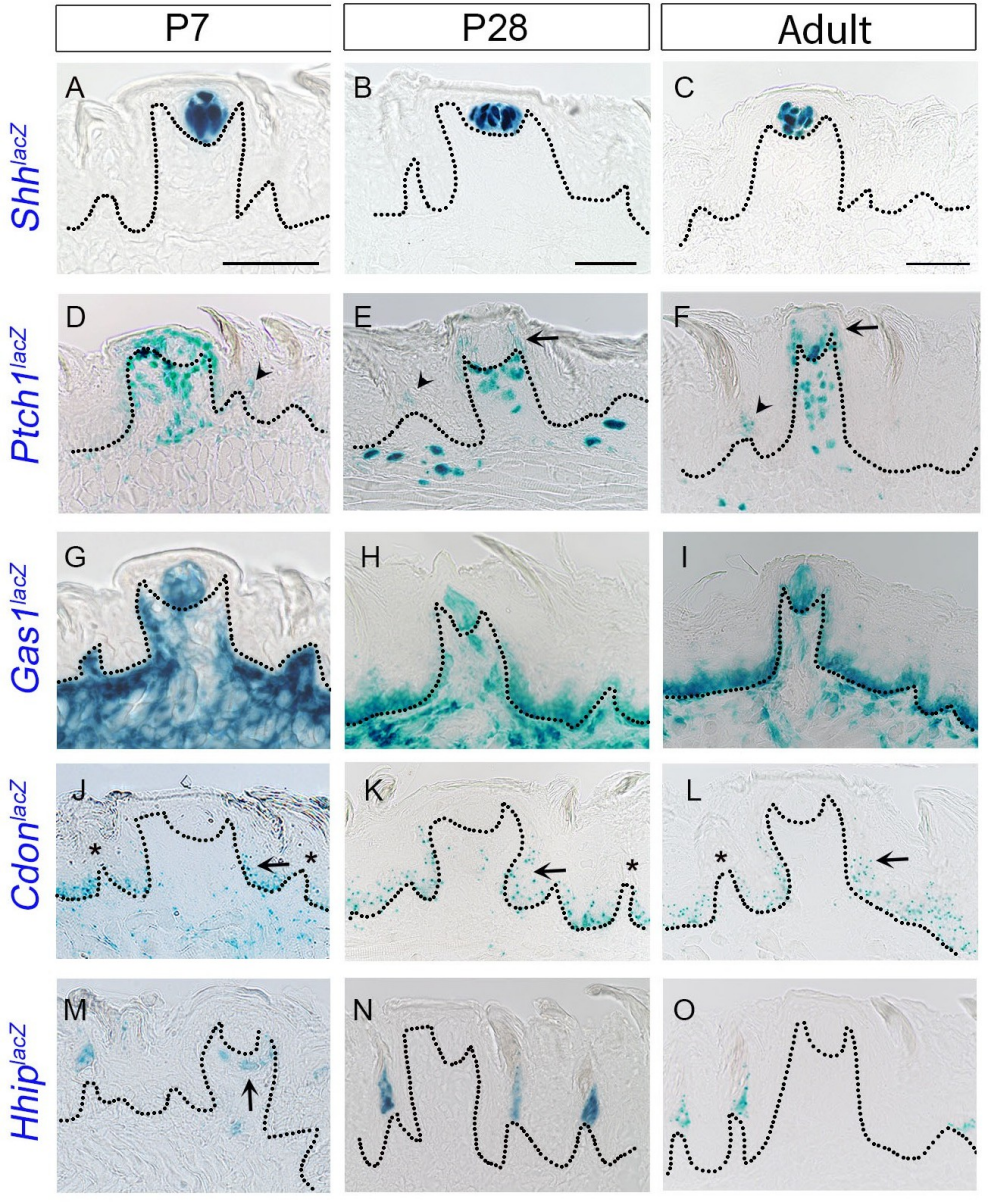
Expression of HH pathway ligand and receptors in the anterior tongue from early postnatal to adult stages. (A-O) X-gal staining using *Shh*^*lacZ*/+^, *Ptch1*^*lacZ/+*^, *Gas1*^*lacZ/+*^, *Cdon*^*lacZ/+*^ and *Hhip*^*lacZ/+*^ reporter mice at P7, P28 and adult stages. *Shh* ligand is expressed within taste buds at P7 (A), P28 (B) and adult tongue (C). *Ptch1*^*lacZ*^ is expressed in the FP epithelium at all stages (D-F), but is predominant in the apical region (E, F arrows) from late postnatal to adulthood. In addition, *Ptch1*^*lacZ*^ is expressed in filiform papillae (FILIF) epithelium (D-F, arrowheads) and tongue stromal cells. *Gas1*^*lacZ*^ is expressed in taste buds at all stages (G-I), extensively in the stroma at P7 (G) and additionally in the epithelium from P28, albeit with less extensive stromal expression (H,I). *Cdon*^*lacZ*^ is observed in the entire lingual epithelium, within FP, it is concentrated in the lower half of the epithelium (J-L, arrows) and within FILIF, it appears not to overlap with *Ptch1* locations (J-L, asterisks). Stromal *Cdon*^*lacZ*^ expression is also decreased from P7 to adult stages (J-L). The HH antagonist *Hhip*^*lacZ*^ is observed in FILIF at all the stages (M-O). *Hhip*^*lacZ*^ expression is also observed in the FP connective tissue core at P7 (M, arrow). Dotted lines outline the epithelium in all images. Scale bars (50μm) in A, B and C apply to respective column images.

In **Fig 1A** specific cell types in FP are illustrated. We compared expression patterns of HH pathway components across three developmental stages (early postnatal at P7, late postnatal at P28 and adult between 8–15 weeks of age). *Shh* ligand is present in FP taste buds, including taste bud basal cells, as observed with X-gal staining of the *Shh*^*lacZ*^ reporter mouse model at P7, P28 and adult tongues (**Fig 2A–2C**) and align with previous studies using the SHH antibody detection method [25–27]. We do not observe ligand expression in nerves with X-gal staining as reported with transgenic reporter models for *Shh* [26,45–47].

The HH membrane receptor *Ptch1* is expressed in both taste FP and non-taste FILIF from P7 through adult stages, as indicated by X-gal staining (**Fig 2D–2F**). However, the pattern in FP shows a shift between early and late postnatal stages. In FP at P7, *Ptch1*^*lacZ*^ expression is observed in taste buds, perigemmal, apical extragemmal, basal and stromal cells (**Fig 2D**). At P28, there is no *Ptch1*^*lacZ*^ expression within taste buds and expression in basal epithelial cells is limited to apical regions of FP, next to perigemmal cells (**Fig 2E**, arrow), while perigemmal, apical extragemmal and stromal cell expression are maintained (**Fig 2E**). This restricted basal cell pattern is also observed in the adult stage (**Fig 2F**, arrow). Upon investigating an earlier stage, P19, we again find a reduction in *Ptch1*^*lacZ*^ expression in FP basal cells and loss of expression within taste buds (**S1A Fig**). In contrast, *Ptch1*^*lacZ*^ expression in FILIF anterior epithelial face in cells of the basal and suprabasal layers is maintained from early postnatal stages to adulthood (**Figs 2D–2F**, arrowheads; **S1B**).

X-gal staining of *Gas1*^*lacZ*^ mouse tongue revealed expression in FP taste buds from P7 to adult stages (**Fig 2G–2I**). While no other epithelial *Gas1*^*lacZ*^ expression is present at P7 (**Figs 2G and S1C**, Ecad), expression emerges in the basal lingual epithelium on and after P21 (**Figs 2H and 2I and S1D and S1E**). In FP, *Gas1*^*lacZ*^ expression is virtually absent in perigemmal and apical extragemmal cells, and the pattern is maintained through the adult stage (**Fig 2G–2I**). Further, we find extensive *Gas1*^*lacZ*^+ cells in the lingual stroma (**Fig 2G**) and muscles (**S1C Fig**) at or before P7. However, stromal expression declines while expression in muscle ceases on and after P21 (**Figs 2H and 2I** and **S1D and S1E**). We find that *Gas1*^*lacZ*^ expression changed between P7 and P21, as indicated by a reduction in stromal cells, elimination in muscle cells and simultaneous emerging expression in lingual epithelial cells.

Another membrane HH co-receptor, *Cdon*^*lacZ*^, is expressed in the entire lingual basal epithelium (but not in the apical half of FP wall) from P7 to the adult stage (**Fig 2J–2L**). This is unlike other HH receptors, *Ptch1* and *Gas1*, which change their lingual epithelial expression from early postnatal stages to adulthood. Intriguingly, in FP, *Cdon*^*lacZ*^ expression is predominant in the basal lower half of FP (**Fig 2J–2L,** arrows) and in FILIF appears not to overlap with *Ptch1* expression (**Fig 2J–2L,** asterisks). Although epithelial *Cdon*^*lacZ*^ expression in FP and FILIF is retained, its expression in overall tongue stromal cells showed reduction at eight weeks of age as compared to P7 or P28 (**Figs 2J–2L and S1F and S1G**). There is no *Cdon* ^*lacZ*^ expression in taste buds, perigemmal and apical extragemmal cells in any of the tongue developmental stages (**Figs 2J–2L and S1F and S1G**).

Similar to our recent study [27], HH antagonist *Hhip*^*lacZ*^ expression is observed in FILIF apical cells, consistently from P7 to adult stages (**Fig 2M–2O**). We do not observe *Hhip*^*lacZ*^ expression in taste buds, perigemmal and apical extragemmal cells (**Fig 2M–2O**). We observe expression of *Hhip*^*lacZ*^ in a few stromal cells within FP connective tissue core concentrating below the taste buds and in tongue stromal cells at P7. Stromal *Hhip* expression is noted at P12 (**S1H Fig** arrows) but not at P28 and adult stage (**Figs 2N and 2O and S1I**).

Overall, the data indicate that all transmembrane HH-receptors are expressed in the anterior tongue with partial to no co-expression, suggesting distinct and non-overlapping function in the development and maintenance of taste and non-taste organs. Intriguingly, we observed shifts in the unique expression patterns of *Ptch1*, *Gas1* and *Hhip* in the early postnatal period primarily between P7-P21, suggesting a shift in function or a multi-functional role. Among the four HH membrane receptors studied here, only *Gas1*^*lacZ*^ is consistently present in taste buds.

### Transcription factor *Gli1* expression changes during initial postnatal weeks, whereas *Gli2* and *Gli3* expressions remain stable in anterior tongue

**Fig 3:**
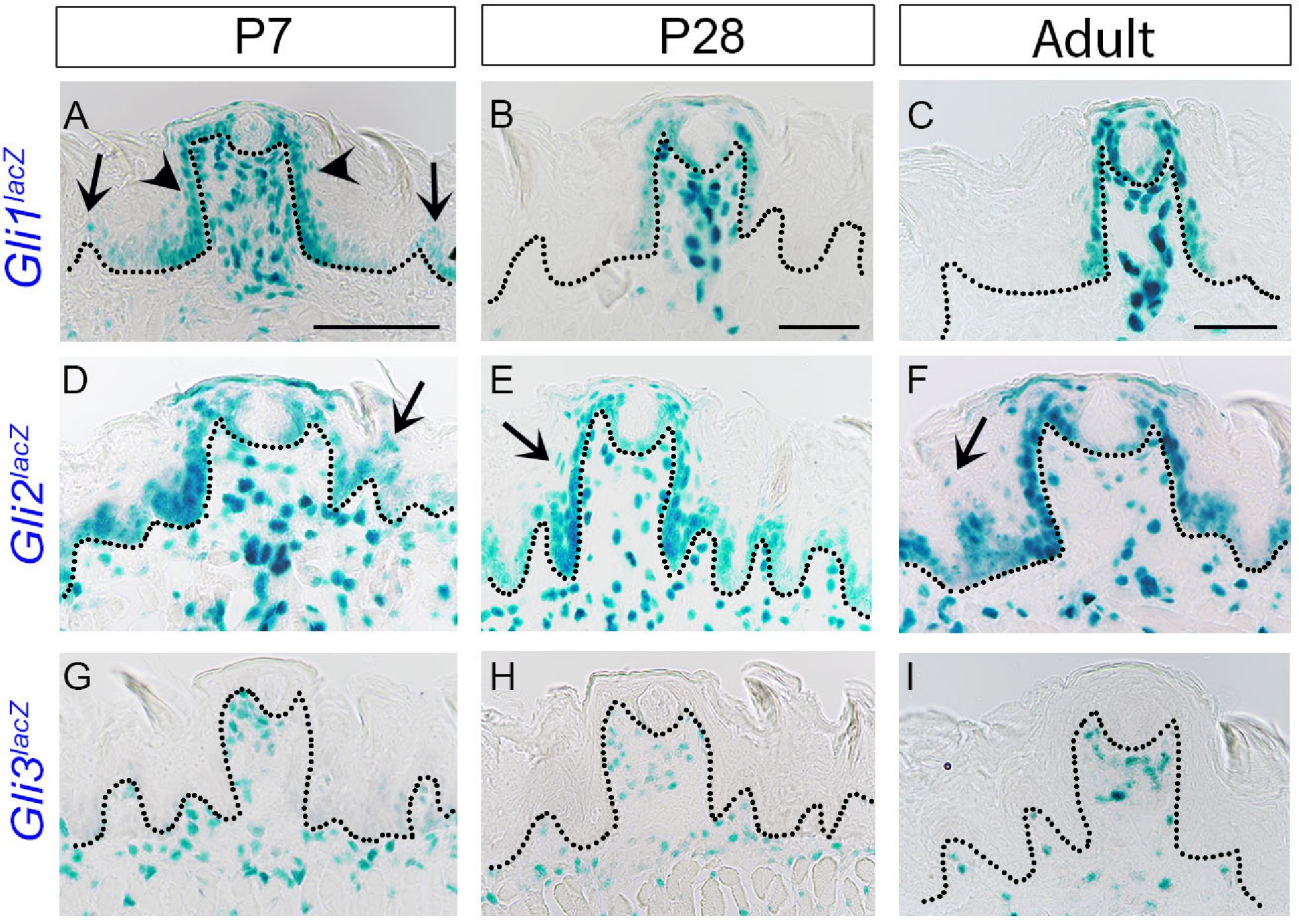
HH pathway transcription factors expression in the anterior tongue from early postnatal to adult stages. (A-I) X-gal staining of *Gli1*^*lacZ/+*^, *Gli2*^*lacZ/+*^and *Gli3*^*lacZ/+*^ reporter mice at the P7, P28 and adult stages. *Gli1*^*lacZ*^ is expressed in FP basal, perigemmal, apical extragemmal and stromal cells (A-C). In addition, at P7, *Gli1*^*lacZ*^ is observed in FILIF (A, arrows) and FP suprabasal epithelial layers (A, arrowheads). *Gli2*^*lacZ*^ expression is observed in the entire lingual basal epithelium, suprabasal layers (D-F, arrows), perigemmal, apical extragemmal and stromal cells in (D-F). In contrast to *Gli1*^*lacZ*^ and *Gli2*^*lacZ*^, *Gli3*^*lacZ*^ is only present in lingual stromal cells (G-I). Dotted lines outline the epithelium in all images. Scale bars (50μm) in A,B and C apply to respective column images.

As reported earlier [9,25–27,48], *Gli1*^*lacZ*^ expression is seen in FP basal, perigemmal, apical extragemmal and stromal cells and is maintained throughout postnatal and adult stages (**Fig 3A–3C**). However, we additionally observed *Gli1*^*lacZ*^*+* cells in FILIF at P7 (**Fig 3A**, arrows), which are not seen after P28 (**Fig 3B and 3C**). To determine when *Gli1* expression becomes restricted in the FP, we stained *Gli1*^*lacZ*^ mouse tongues from a later developmental timepoint (P14). We found that 50% of FP lost *lacZ* expression in neighboring FILIF (**S1J Fig**). After P28, *Gli1*^*lacZ*^+ cells are observed only in FP (**Fig 3B and 3C**). Further, the expression is noted in basal and a few suprabasal cells at P7 (**Fig 3A**, arrowheads) but is restricted to FP basal cells only after P28 (**Fig 3B and 3C**).

*Gli2* reporter gene expression is observed in the entire lingual basal epithelium, perigemmal, apical extragemmal cells (including both FP and FILIF), and also in stromal cells from P7 to adult stages in *Gli2*^*lacZ*^ mice (**Fig 3D–3F**). In addition, a few suprabasal epithelial cells of both FP and FILIF show *Gli2*^*lacZ*^ expression (**Fig 3D–3F**, arrows). Stromal *Gli2*^*lacZ*^ expression in the tongue is extensive as compared to *Gli1*^*lacZ*^ expression throughout all stages (**Fig 3D–3F**). There is no overt change or shift in the expression pattern of *Gli2*^*lacZ*^ in the adult tongue as compared to the early postnatal stages.

X-gal staining of *Gli3*^*lacZ*^ mouse tongues revealed positive cells throughout the lingual stroma in both FP and FILIF, which remained unaltered from P7 to adult stages (**Fig 3G–3I**). Unlike other *Gli* transcription factors, *Gli3* is not expressed in any of the epithelial (basal, perigemmal and apical extragemmal) cells. We found faint *Gli3*^*lacZ*^ expression in a few taste buds in the adult stage after 48 hours of X-gal staining. A previous study in adult mice reported *Gli3*+ taste buds cells by *in situ* hybridization using digoxigenin-labeled *Gli3* RNA probes [49].

The data suggest somewhat overlapping patterns of epithelial expression of *Gli1* and *Gli2* in FP and FILIF during initial postnatal stages. Similar *Gli1* and *Gli2* expression in FP are retained in late postnatal through adult stages. In contrast, *Gli3* is observed only in stromal cells. Whether there is any overlap between *Gli* stromal expressions was not studied.

### Mapping *Shh* ligand and membrane receptors *Ptch1*, *Gas1*, *Cdon* and *Hhip* expression in posterior circumvallate and foliate papillae at early postnatal and adult stages

**Fig 4:**
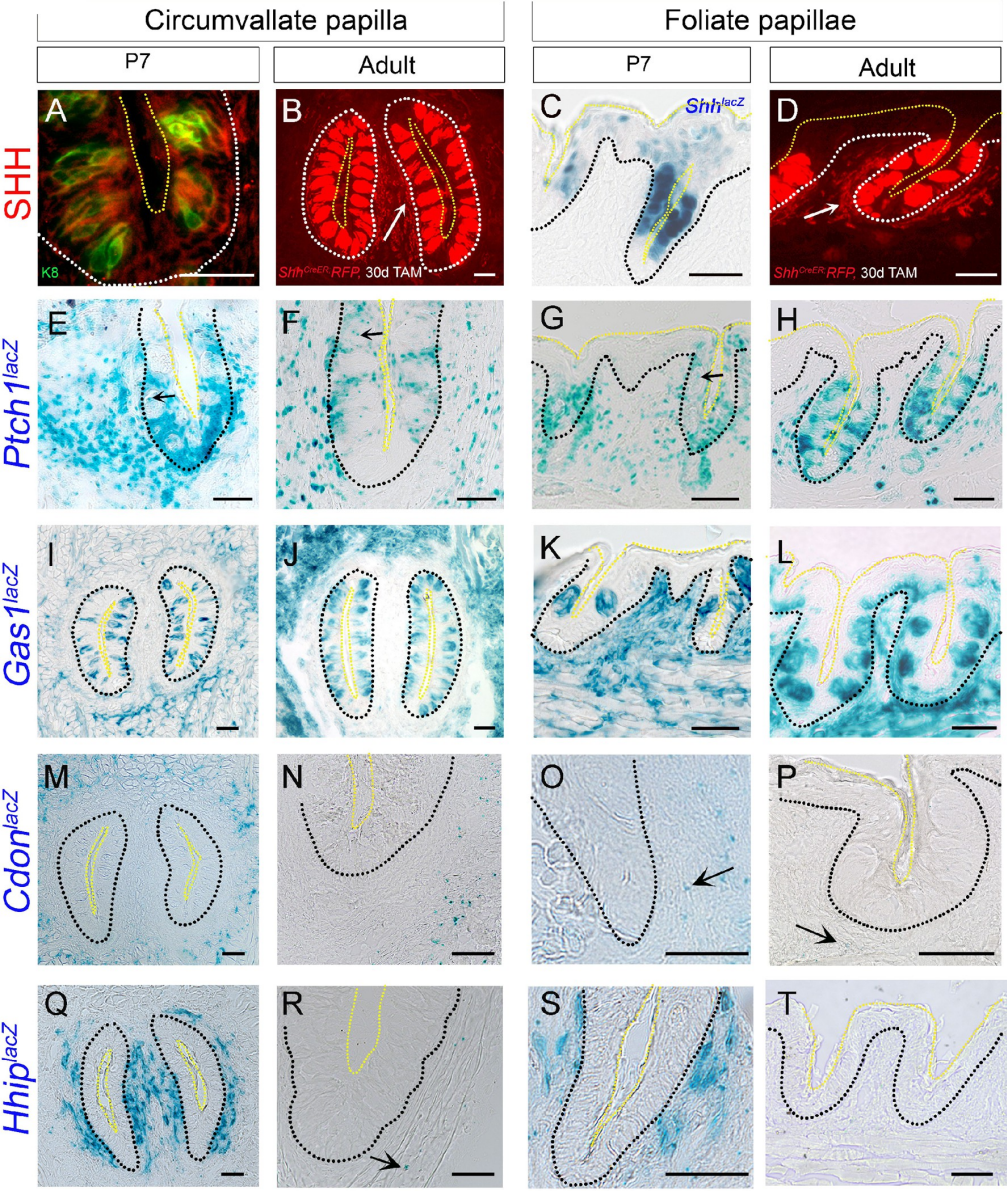
Expression of HH pathway ligand and receptors in posterior tongue circumvallate and foliate papillae from early postnatal and adult stages. (A-D) SHH ligand expression within CVP and FOP taste buds (labelled with K8, green, A) as demonstrated by either antibody staining or X-gal staining in *Shh*^*lacZ*/+^ reporter mouse at P7 (A,C) and RFP staining in *Shh*^*CreERT2*^*;R26*^*RFP*^ reporter mouse after 30 days of tamoxifen treatment at adult stage (B,D).(E-T) X-gal staining using *Shh*^*lacZ*/+^, *Ptch1*^*lacZ/+*^, *Gas1*^*lacZ/+*^, *Cdon*^*lacZ/+*^ and *Hhip*^*lacZ/+*^ reporter mice at P7 and adult stages. In both stages, *Ptch1*^*lacZ*^ is expressed in basal, perigemmal, apical extragemmal and stromal cells of CVP (E, F) and FOP (G,H). A few *Ptch1*^*lacZ*^ punctae are seen within taste buds of CVP at both stages (E, F, arrows) and FOP at P7 (G, arrow). *Gas1*^*lacZ*^ is present in taste buds and stromal cells at P7 and in adult CVP (I, J) and FOP (K, L). Stromal expression of *Cdon*^*lacZ*^ is observed in CVP (M,N) and FOP (O,P), but the expression declines at adult stages (N,P) as compared to P7 (M,O). Extensive *Hhip*^*lacZ*^ CVP and FOP stromal expression is observed at P7 (Q,S), which is virtually eliminated by the adult stage (R,T), and few punctate *lacZ*+ cells are observed in CVP stroma at adult stage (R, arrow). Black or white dotted lines outline the epithelium and yellow dotted lines outline the taste buds apical surface in all the images. Scale bars are 50μm.

**Fig 5:**
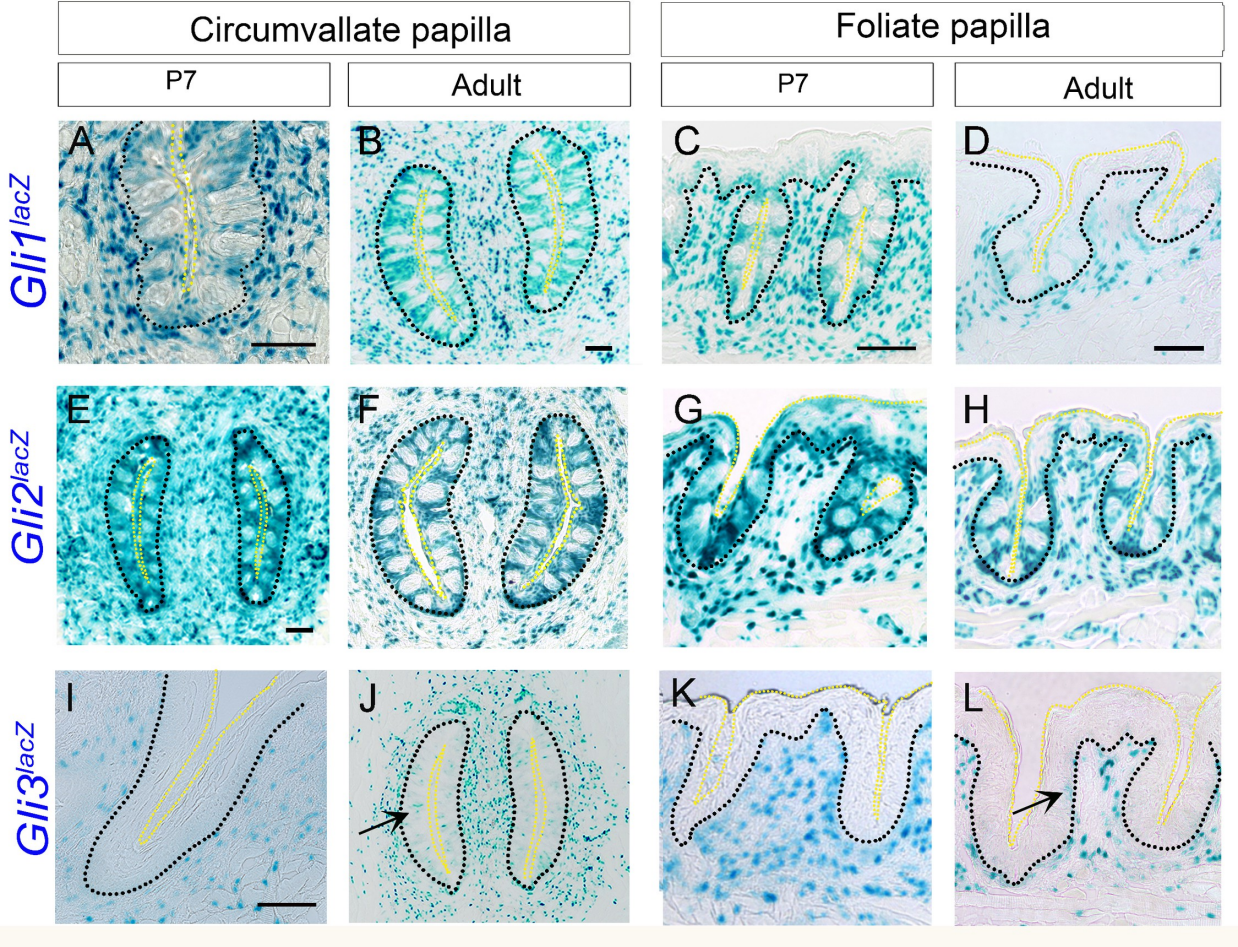
Expression pattern of HH pathway transcription factors in circumvallate and foliate papillae from early postnatal and adult stages. (A-L) X-gal staining of *Gli1*^*lacZ/+*^, *Gli2*^*lacZ/+*^and *Gli3*^*lacZ/+*^ reporter mice at P7 and adult stages. *Gli1*^*lacZ*^ (A-D) and *Gli2*^*lacZ*^ (E-H) are expressed in basal, perigemmal, apical extragemmal and stromal cells of CVP (A,B,E,F) and FOP (C,D,G,H) at both the P7 and adult stages. *Gli3*^*lacZ*^ is present in CVP and FOP stromal cells (I-L) at P7 (sagittal section) and adult tongue (horizontal section) and additionally in taste buds in adult CVP and FOP taste buds (J,L arrows). Black dotted lines outline the epithelium and yellow dotted lines outline the taste buds apical surface in all the images. Scale bars (50μm) in B,C and D apply to respective column images.

Illustrations for CVP and FOP structure and cell types in the horizontal plane are shown in **Fig 1A**. Given the continuous postnatal development of posterior CVP and FOP taste buds [10], we studied the initial postnatal week with limited taste bud formation and mature adult stages with a full complement of taste buds to analyze HH pathway component expression (**Figs 4 and 5**). Immunostaining with SHH antibody or X-gal staining of the *Shh*^*lacZ*^ reporter mouse model at P7 showed ligand expression in CVP and FOP taste buds (**Figs 4A and 4C and** K8+; **S1K**). To visualize *Shh* expression in nerves, we used tissues from *Shh*^*CreERT2*^*; R26*^*RFP*^ animals to determine whether nerves are labeled in adult CVP and FOP. After 30 days of tamoxifen induction *Shh* expression is observed in nerve fibers surrounding the CVP walls and all FOP ridges in the connective tissue core (**Fig 4B and 4D**, arrows) in addition to taste buds. Previous work using the *Shh*^*CreER/+*^*; R26*^*mTmG*^ model also showed *Shh*+ fibers within CVP taste buds [47]. CVP and the posterior-most folds of the FOP are innervated by glossopharyngeal nerves (**Fig 1A**), which have soma in the petrosal ganglion. *Shh* expression in the petrosal ganglion is not yet studied.

For *Ptch1*^*lacZ*^ expression, we investigated CVP and FOP sagittal sections at P7 and horizontal sections at the adult stage (**Fig 4E–4H**). We observed *Ptch1*^*lacZ*^+ cells in CVP and FOP basal, perigemmal, apical extragemmal and stromal cells at both stages (**Fig 4E–4H**). Magnification of the CVP wall shows few punctate *Ptch1*^*lacZ*^+ cells within taste buds at either P7 or adult stages (**Fig 4E and 4F**, arrows). Little *Ptch1*^*lacZ*^ expression is seen within FOP taste buds at P7 (**Fig 4G**, arrow).

X-gal staining of horizontal sections of the *Gas1*^*lacZ*^ mouse posterior tongue at P7 and adult revealed expression in CVP and FOP taste buds (**Fig 4I–4L**). There is extensive *Gas1*^*lacZ*^ expression in CVP stroma, but stromal cells of CVP adjacent to papilla walls had few *Gas1*^*lacZ*^+ cells as compared to overall *Gas1*^*lacZ*^ CVP stromal expression at both the stages (**Fig 4I and 4J**). *Gas1*^*lacZ*^ is expressed throughout FOP stroma (**Fig 4K and 4L**).

Contrasting with *Gas1*, *Cdon*^*lacZ*^ expression is not seen in either CVP or FOP taste buds (**Fig 4M–4P**). The *Cdon*^*lacZ*^+ stromal cell expression pattern at P7 in the CVP connective tissue core is comparable to the *Gas1*^*lacZ*^ arrangement, with few positive cells surrounding papilla walls whereas the expression is prominent in the rest of the stromal cells distant to papilla walls (**Fig 4M**). We observed *Cdon*^*lacZ*^+ cells throughout FOP stromal cells at P7 (**Fig 4O**). In both CVP and FOP at adult stages (**Fig 4N and 4P**), the expression declined as compared to P7 (**Fig 4M and 4O**). It is possible that the remaining punctate X-gal staining represents residual β-gal enzyme rather than active gene expression [50]. Unlike the anterior FP expression pattern (**Fig 1J–1L**), we do not observe *Cdon*^*lacZ*^ expression in CVP or FOP basal cells (**Fig 4M–4P**).

In contrast to *Gas1*^*lacZ*^ and *Cdon*^*lacZ*^ expression patterns in the connective tissue core, *Hhip*^*lacZ*^ is extensively expressed in CVP and FOP stromal cells neighboring the papilla walls at P7 (**Fig 4Q and 4S**). Further, *Hhip*^*lacZ*^ expression is limited to a few stromal cells in adult CVP and virtually absent in the adult FOP connective tissue core, as indicated in horizontal sections (**Fig 4R,** arrow; **4T**). The stromal CVP and FOP *Hhip* expression at an early developmental stage (**Fig 4Q and 4S**) is coincident with expression in FP stroma (**S1H Fig**).

Overall, the expression patterns of *Shh* ligand and HH membrane receptors *Ptch1*, *Gas1*, and *Cdon* remain unchanged during the postnatal development of CVP and FOP. Only *Cdon* and *Hhip* expression changed in the CVP and FOP connective tissue core at 8 weeks of age as compared to the first postnatal week. Interestingly, basal epithelial expression of *Gas1* and *Cdon*, as seen in anterior tongue, is not observed in the posterior CVP and FOP at any given developmental stage(s). The data indicate the requirement of different HH receptors between anterior and posterior tongue epithelial development.

### *Gli* transcription factor expression in the posterior circumvallate and foliate papillae remain the same throughout postnatal development, while *Gli3* is additionally expressed in papilla taste buds in adults

In both P7 and adult tongues, we observed *Gli1*^*lacZ*^ expression in CVP and FOP basal, perigemmal, apical extragemmal and stromal cells (**Fig 5A–5D**). Similar to *Gli1*^*lacZ*^, *Gli2*^*lacZ*^+ cells are present in CVP and FOP basal, perigemmal, apical extragemmal and stromal cells throughout postnatal and adult stages (**Fig 5E–5H**). There is no *Gli1*^*lacZ*^ or *Gli2*^*lacZ*^ expression within taste buds of CVP or FOP (**Fig 5A–5H**).

X-gal staining of P7 sagittal tongue sections revealed *Gli3*^*lacZ*^+ cells in CVP and FOP stroma (**Fig 5I and 5K**). *Gli3*^*lacZ*^ expression in CVP and FOP stroma surrounding papilla walls remains similar in adult horizontal tongue sections (**Fig 5J and 5L**) as compared to P7 (**Fig 5I–5K**). In addition, there are positive cells in CVP and FOP taste buds in mice older than 8 weeks (**Fig 5J and 5L,** arrows).

The data indicate that *Gli* transcription factor expression in CVP and FOP mostly remains the same from early postnatal weeks through adult stages, and the location patterns are similar to each other and to the anterior tongue.

### HH signaling component expression in adult soft palate is similar to fungiform papilla

**Fig 6:**
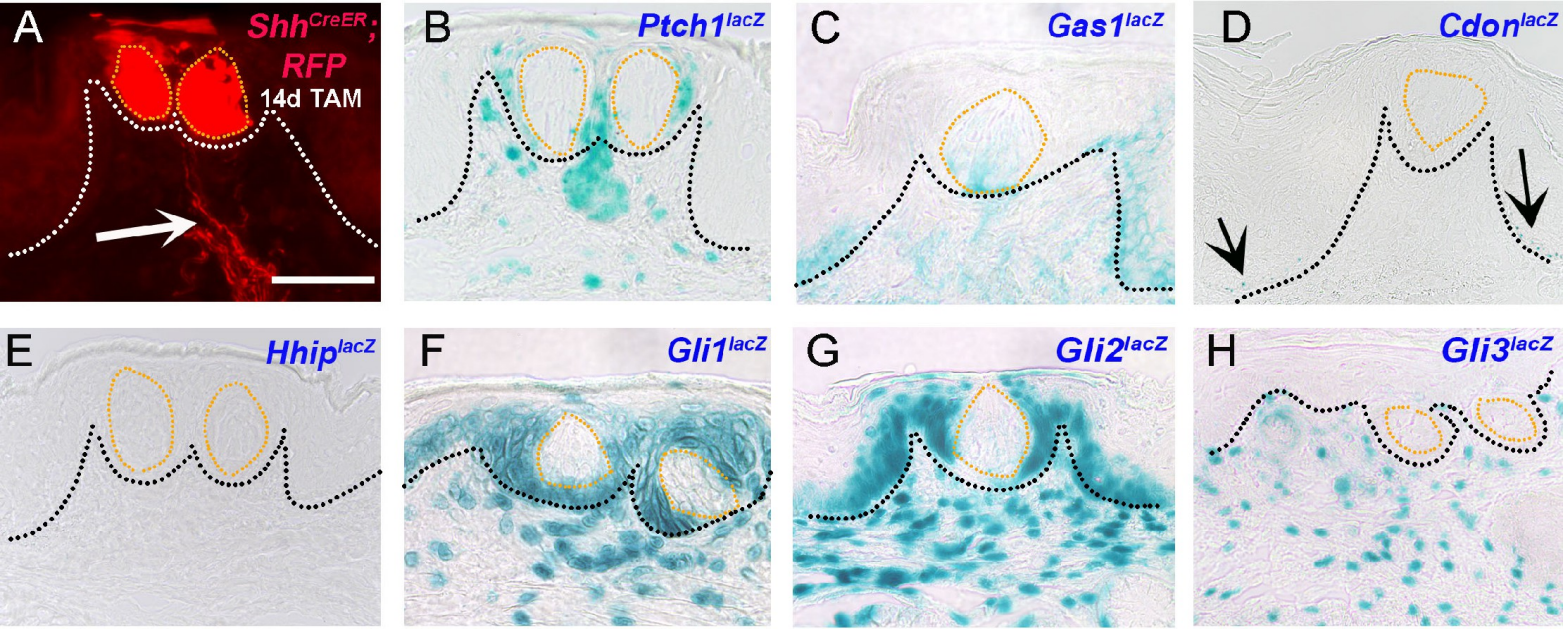
Expression of HH pathway components in adult mouse soft palate. (A) RFP staining in *Shh*^*CreERT2*^*;R26*^*RFP*^ reporter mouse after 14 days of tamoxifen treatment reveals *Shh* expression in the soft palate (SP) taste buds and nerves (A, arrow). (B) *Ptch1*^*lacZ*^ is expressed in perigemmal and stromal cells along with punctate expression within taste buds. (C) *Gas1*^*lacZ*^ is present within the taste buds, epithelium and stroma. (D) *Cdon*^*lacZ*^ expression is seen at the SP epithelium rete ridges (D, arrows). (E) *Hhip*^*lacZ*^ is not expressed in the SP. (F-G) Both *Gli1*^*lacZ*^ (F) and *Gli2*^*lacZ*^ (G) are present in SP basal, perigemmal, apical extragemmal and stromal cells. (H) *Gli3*^*lacZ*^ is exclusively present in the stromal cells. Black or white dotted lines outline the epithelium and orange dotted lines outline the taste buds in all the images. Scale bar (50μm) in A applies to all images.

We investigated the soft palate (SP) with taste organs in the oral cavity that harbor taste buds distributed in the epithelium, not enclosed in papillae. We limited our investigations to the adult stage. X-gal staining of the *Shh*^*lacZ*^ mouse SP reveals expression in taste buds (**S1L Fig**) consistent with previous findings [51]. Further, with the 14-day tamoxifen-induced *Shh*^*CreERT2*^*;R26*^*RFP*^ mouse model, *Shh* is observed in both taste buds and nerves (**Fig 6A**). Different transmembrane HH receptors have distinct expression: *Ptch1*^*lacZ*^ in SP perigemmal and stromal cells with some punctate expression within taste buds cells (**Fig 6B**); *Gas1*^*lacZ*^ in SP basal epithelial, stromal and taste buds cells (**Fig 6C**); *Cdon*^*lacZ*^ in the SP basal epithelium, mainly in the lower half (**Fig 6D**). We did not observe *Hhip*^*lacZ*^ expression in SP (**Fig 6E**) as reported earlier [27]. All HH transcription factors *Gli1*^*lacZ*^, *Gli2*^*lacZ*^ and *Gli3*^*lacZ*^ are expressed within SP stromal cells (**Fig 6F–6H**). *Gli1*^*lacZ*^ and *Gli2*^*lacZ*^ are additionally expressed in SP basal epithelial, perigemmal and apical extragemmal cells (**Fig 6F and 6G**). Our HH component expression data reveal active HH signaling in SP, similar to FP.

### SHH ligand, membrane receptor *Ptch1*, and transcription factors *Gli1* and *Gli2* are the only HH pathway components expressed in adult geniculate and trigeminal ganglia

**Fig 7:**
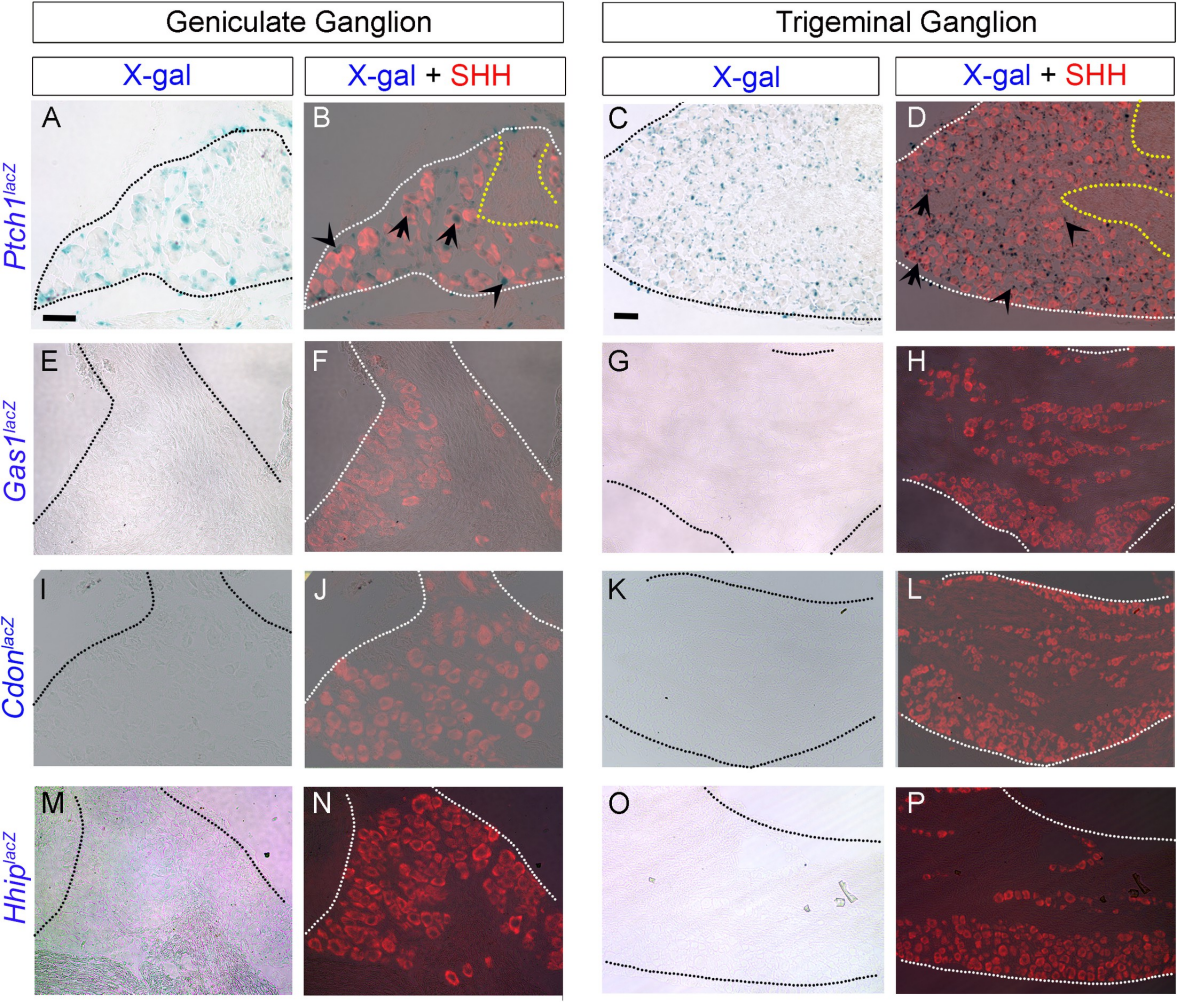
Expression pattern of HH ligand and receptors in adult mouse geniculate and trigeminal ganglia. (A-P) X-gal staining in *Ptch1*^*lacZ/+*^, *Gas1*^*lacZ/+*^, *Cdon*^*lacZ/+*^ and *Hhip*^*lacZ/+*^ reporter mice. SHH antibody staining indicates SHH+ (red) cell bodies in GG (B, F, J, N) and TG (D, H, L, P). *Ptch1*^*lacZ*^ expression is observed both in GG (A, B) and TG (C, D) and overlaps (arrows) or neighbors (arrowheads) SHH+ cells (B, D red). Yellow lines demarcate nerve bundles exiting the ganglion. None of the other HH-co-receptors, *Gas1*^*lacZ*^ (E-H), *Cdon*^*lacZ*^ (I-L) and *Hhip*^*lacZ*^ (M-P) is expressed in either GG or TG. Scale bars (50μm) in A and C apply to all Geniculate Ganglion and Trigeminal Ganglion images, respectively.

Geniculate ganglion (GG) and trigeminal ganglion (TG) contain cell bodies of chorda tympani and lingual nerves, respectively, that innervate the anterior tongue [52]. Previous studies with reporter mice indicate *Shh* ligand expression in the adult mouse GG [26,45,47] and TG [26]. Here, we have used a SHH antibody and reproduced the finding of SHH+ soma in both GG (**Fig 7B, 7F, 7J and 7N**) and TG (**Fig 7D, 7H, 7L and 7P**). Among all the HH co-receptors, *Ptch1* is the only one expressed in both GG and TG (**Fig 7A–7D**). We analyzed *Ptch1* expression qualitatively based on location within the neuron and outside the neuron, touching either the cell body or the nerve bundles exiting the ganglion. Our data indicate that the *Ptch1*^*lacZ*^+ cells either overlap (arrows) or are adjacent to (arrowheads) SHH+ neuronal cells in adult GG and TG (**Fig 7B and 7D**). There is no expression of *Gas1*^*lacZ*^ (**Fig 7E–7H**), *Cdon*^*lacZ*^ (**Fig 7I–7L**) or *Hhip*^*lacZ*^ (**Fig 7M–7P**) in either GG or TG.

**Fig 8:**
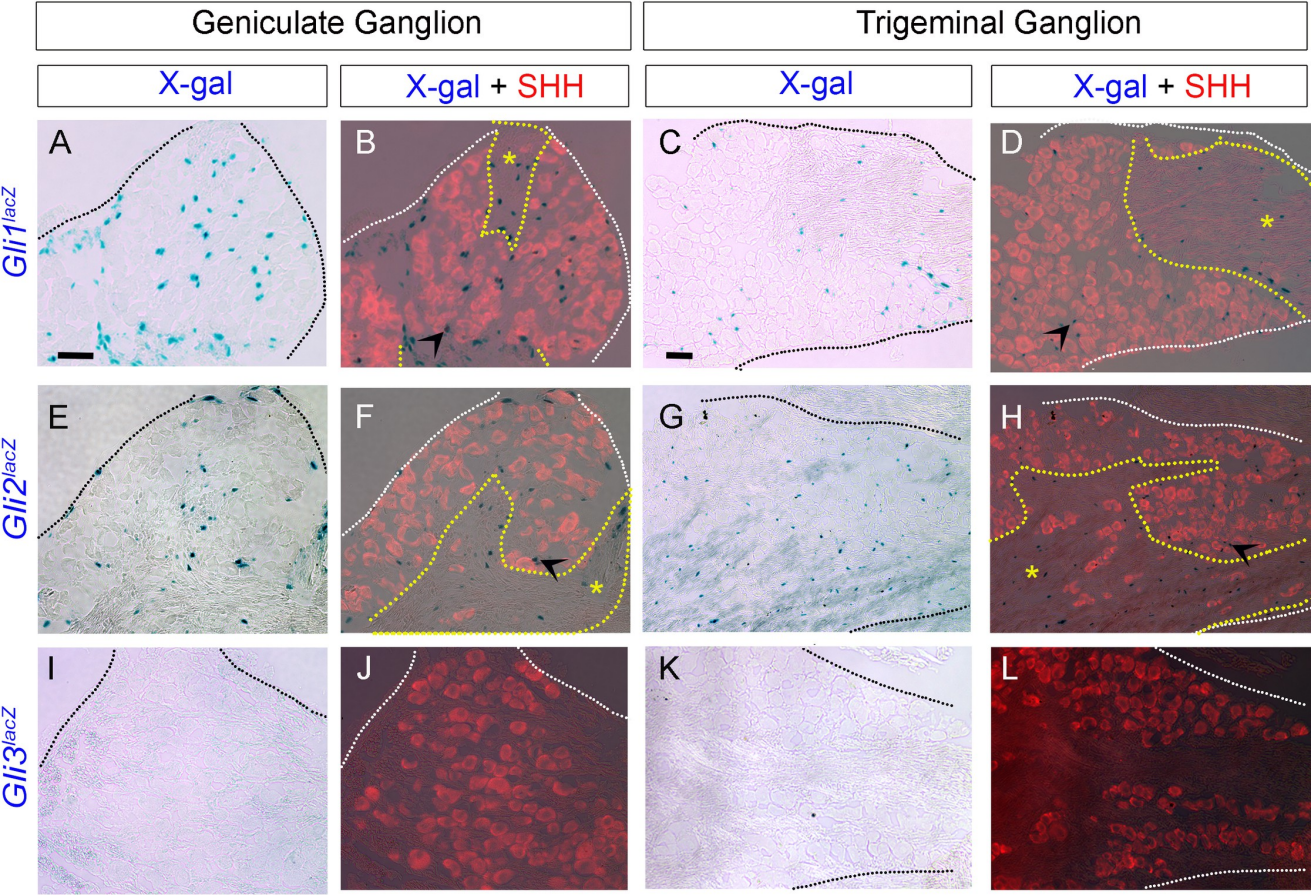
Expression pattern of HH transcription factors in adult mouse geniculate and trigeminal ganglia. (A-L) X-gal staining in *Gli1*^*lacZ/+*^, *Gli2*^*lacZ/+*^ and *Gli3*^*lacZ/+*^ reporter mice. SHH antibody staining indicates SHH+ (red) cell bodies in GG (B, F, J) and TG (D,H,L). Both *Gli1*^*lacZ*^ (A-D) and *Gli2*^*lacZ*^ (E-H) expressions are observed in GG (A,B,E,F) and TG (C,D,G,H) with (arrowheads) and without neighboring SHH ligand (B,D,F,H red). Additionally, both are expressed within the nerve bundles exiting the ganglion (B, D, F, H, asterisks). Yellow lines demarcate nerve bundles exiting the ganglion. In contrast, the transcription factor *Gli3*^*lacZ*^ is not expressed in either GG or TG (I-L). Scale bars (50μm) in A and C apply to all Geniculate Ganglion and Trigeminal Ganglion images, respectively.

Among the transcription factors, *Gli1*^*lacZ*^ and *Gli2*^*lacZ*^, but not *Gli3*^*lacZ*^ are observed in both GG and TG (**Fig 8A–8L**). Further, *Gli1*^*lacZ*^+ and *Gli2*^*lacZ*^+ cells are either next to SHH+ cells (arrowhead) or within the nerve bundles exiting the ganglion (asterisk) (**Fig 8B, 8D, 8F and 8H**). Importantly, in our observations not all SHH+ neurons have adjacent *Gli1*^*lacZ*^+ and *Gli2*^*lacZ*^+ cells suggesting specificity towards a neuron cell type for a particular function.

Overall, the presence of SHH, *Ptch1*, *Gli1* and *Gli2* indicates a requirement for active HH signaling in GG and TG while the absence of *Gas1*, *Cdon*, *Hhip* and *Gli3* implies that there is no HH antagonism in either ganglion.

## Discussion

Here we have analyzed the expression of HH pathway ligand, membrane receptors and antagonist, and the GLI transcription factors in the gustatory system during development and/or homeostasis. We have examined HH pathway component expressions across six distinct tissues, FP, CVP, FOP, SP, GG, and TG (**Table 1**). Our data unveil unique expression patterns in the lingual epithelium, stroma and muscles. The identified shifts in gene expressions during late postnatal stages match with the conclusion of anterior tongue morphogenesis by postnatal day 21 [10,15,16,18]. Further, the HH pathway receptors, *Gas1* and *Cdon*, which remain unexplored in the tongue, have extensive yet dynamic lingual epithelium, stromal and muscular expressions.

**Table 1:** Summary of HH pathway component expressions in gustatory system.

		*Ligand*	*Receptors*				*Transcription factors*		
		*Shh*	*Ptch1*	*Gas1*	*Cdon*	*Hhip*	*Gli1*	*Gli2*	*Gli3*
**Taste bud**									
FP	Early postnatal	++	+	++	−	−	−	−	−
	Late postnatal	++	−	++	−	−	−	−	−
	Adult	++	−	++	−	−	−	−	−
CV	Early postnatal	++	+	++	−	−	−	−	−
	Adult	++	+	++	−	−	−	−	+
FOP	Early postnatal	++	+	++	−	−	−	−	−
	Adult	++	−	++	−	−	−	−	+
SP	Adult	++	+	++	−	−	−	−	−
**Basal epithelium**									
FP	Early postnatal	−	++	−	++	−	++	++	−
	Late postnatal	−	+	++	++	−	++	++	−
	Adult	−	+	++	++	−	++	++	−
CV	Early postnatal	−	++	−	−	−	++	++	−
	Adult	−	++	−	−	−	++	++	−
FOP	Early postnatal	−	++	−	−	−	++	++	−
	Adult	−	++	−	−	−	++	++	−
SP	Adult	−	−	+	+	−	++	++	−
**Stroma**									
FP	Early postnatal	−	++	++	++	+	++	++	++
	Late postnatal	−	++	+	+	−	++	++	++
	Adult	−	++	+	−	−	++	++	++
CV	Early postnatal	−	++	++	++	++	++	++	++
	Adult	−	++	++	+	−	++	++	++
FOP	Early postnatal	−	++	++	++	++	++	++	++
	Adult	−	++	++	+	−	++	++	++
SP	Adult	−	++	++	−	−	++	++	++
**Ganglia**									
GG	Adult	++	+	−	−	−	+	+	−
TG	Adult	++	+	−	−	−	+	+	−

Tissues with (–) No, (+) Partial and (++) complete expression distribution.

### *Shh* ligand is present in taste buds and nerves of tongue and soft palate

While previous studies have demonstrated that SHH is expressed in FP taste bud cells and nerves [9,26,45,47,53], data on other taste organs are lacking and focused mainly on the adult stage. Here we show that *Shh* ligand is consistently expressed within the taste buds and in the nerves entering taste buds of all tongue papillae and soft palate (**Table 1**). Whereas nerve-derived HH ligands participate in touch dome [54,55] and hair follicle [56] maintenance, similar effects in postnatal or adult FP taste cell differentiation were not observed [8,45]. The roles of neural SHH on posterior tongue and SP taste buds have not yet been evaluated.

Recently, it has been shown that misexpression of *Shh* in lingual epithelium induces ectopic taste bud formation in FILIF and the number of ectopic taste buds is 3-fold higher when overexpression occurs at P14 as compared to P1 [8]. In this study, our data indicate that expression of the HH receptor *Gas1* is induced in FILIF at later postnatal stages, which may explain the temporal rise in ectopic taste buds reported in the previous study.

### HH signaling is active in anterior taste and non-taste papilla epithelium during early postnatal tongue development

HH signaling, as indicated by the target gene, *Gli1*, in adult tongue is restricted to taste organs [27]. For postnatal stages, a previous study utilized *in situ* staining and showed *Gli1* expression in FP only [9]. However, *in situ* staining might have limitations for detecting lower gene expression [57]. Thus, we utilized *Gli1* reporter mouse models and confirmed *Gli1*+ cells in FP and intriguingly revealed additional expression in FILIF in the initial postnatal weeks. This expression, however, disappears from FILIF by P21 and beyond. Notably, FILIF grow rapidly in the first postnatal week and steadily thereafter until P21, when they reach full maturation [58]. Given that HH/SMO/GLI inhibition at the adult stage did not alter FILIF structure and pattern [27], we propose that HH signaling regulates FILIF maturation and after the conclusion of postnatal morphogenesis by P21, FILIF become HH-independent.

### HH co-receptors are expressed in tongue taste and non-taste papilla epithelium

We recently reported the expression of the HH receptor *Ptch1* in FP and anterior epithelial face of FILIF and suggested dual roles for the receptor: activator in FP and antagonist in FILIF [27]. In addition to PTCH1, there are additional co-receptors, GAS1, BOC and CDON, that help create the SHH gradient during organ development [34,35,59–63] but remain unexplored in the tongue. Here, we have determined both distinct and overlapping spatiotemporal expression patterns of *Gas1* and *Cdon* in the postnatal lingual epithelium, including both FP and FILIF.

Unlike other receptors, extensive *Gas1* expression within the taste bud of all three taste papillae suggests *Gas1*-mediated SHH signaling for taste cell differentiation. *Gas1* expression is seen in the basal lingual epithelium after P21. HH signaling becomes restricted to FP with concurrent expression of *Gas1* in the postnatal lingual epithelium. Initial studies claimed *Gas1* as an antagonist of HH signaling [59,64] while subsequent studies showed positive regulation by *Gas1* [19,20,63]. As *Gas1* function can be ligand-independent [65], we propose that *Gas1* may have distinct functions in taste buds, FP and FILIF cell contexts depending on ligand availability.

*Cdon* expression remains the same in the anterior lingual epithelium throughout postnatal stages, irrespective of the changes in *Gli1* and *Gas1* expression. *Cdon* expression, which co-occurs with *Gli1* in the early postnatal stage and overlaps with *Gas1* in the late postnatal stage, suggests its potential role as a multifunctional regulator of HH signaling during tongue maturation. This role may involve promoting HH signaling in FILIF during early stages and later inhibiting it in collaboration with *Gas1*. Interestingly, this cooperation between *Gas1* and *Cdon* was not observed during limb development [19], even though they are expressed in overlapping domains in the limb [62,63]. Our data further suggest that *Cdon* may not overlap with *Ptch1* suggesting that SHH employs *Cdon*, *Gas1* and *Ptch1* in a context-dependent manner during early and late postnatal stages of tongue development.

### HH co-receptors are differentially expressed in tongue stroma

While previous work has documented *Ptch1* and *Gli* expression in adult lingual stromal cells [9,25,26,48,49], here we identified spatiotemporal expression patterns of two HH co-receptors, *Gas1* and *Cdon* and the antagonist *Hhip* in postnatal lingual stroma (**Table 1**). HH signaling-mediated epithelial–mesenchymal interactions are critical in the development of many tissues, including tooth [66] and palate [67]. In embryonic tongue, stromal cells are involved in the transmission of information from epithelial cells to myogenic progenitor cells for muscle maturation [68,69]; at P14, stromal cells can generate a few taste bud cells [70], but whether they are HH-responsive is unknown. In our epithelial HH/GLI/SMO repression in adult mouse models, stromal cells were retained but could not prevent taste cell loss [25,26], however, this does not rule out a postnatal stromal contribution to FP taste buds.

In limb bud, *Cdon*+ HH responding cells extend long specialized filopodia relaying activation of the pathway at a distance from the cell soma [21]. Similarly, we observed vimentin+ stromal cells with filopodial extensions that contact the basal lamina [25], but whether they are *Cdon*+ is not known. In this study, we observed a decline in HH co-receptor expressions in all tongue papillae stroma at later postnatal stages. Further, the opposing expression patterns of *Hhip* with *Gas1* and *Cdon* in CVP and FOP suggest a discrete population of stromal cells, neighboring and distant to the papillae walls. We hypothesize that stromal HH signaling might provide structural support and regulate the development of lingual epithelium and muscle in the early postnatal stages.

### *Gas1* is the only HH pathway component identified in lingual muscles at early postnatal stage

While HH signaling guides embryonic muscle development [71,72], we found no *Shh* or HH-responding *Gli1* expression in postnatal lingual muscles. This argues against direct HH control of tongue muscles, consistent with other studies suggesting stroma-mediated regulation of muscle development [68,69]. Here, we identified *Gas1* expression in postnatal lingual muscles (**Figs 2G and S1C**) during their crucial maturation phase [73,74]. However, a role for *Gas1* in the postnatal tongue is not yet known. Our recent studies revealed that global *Gas1* deletion did not alter embryonic myoblast migration but caused defects in muscle differentiation leading to altered muscle arrangement [75]. We propose that in addition to its embryonic effect *Gas1* can also have cell-autonomous effects on lingual muscles in early postnatal stages.

### Distinct expression pattern of HH signaling components in anterior and posterior tongue at adult stage

**Fig 9:**
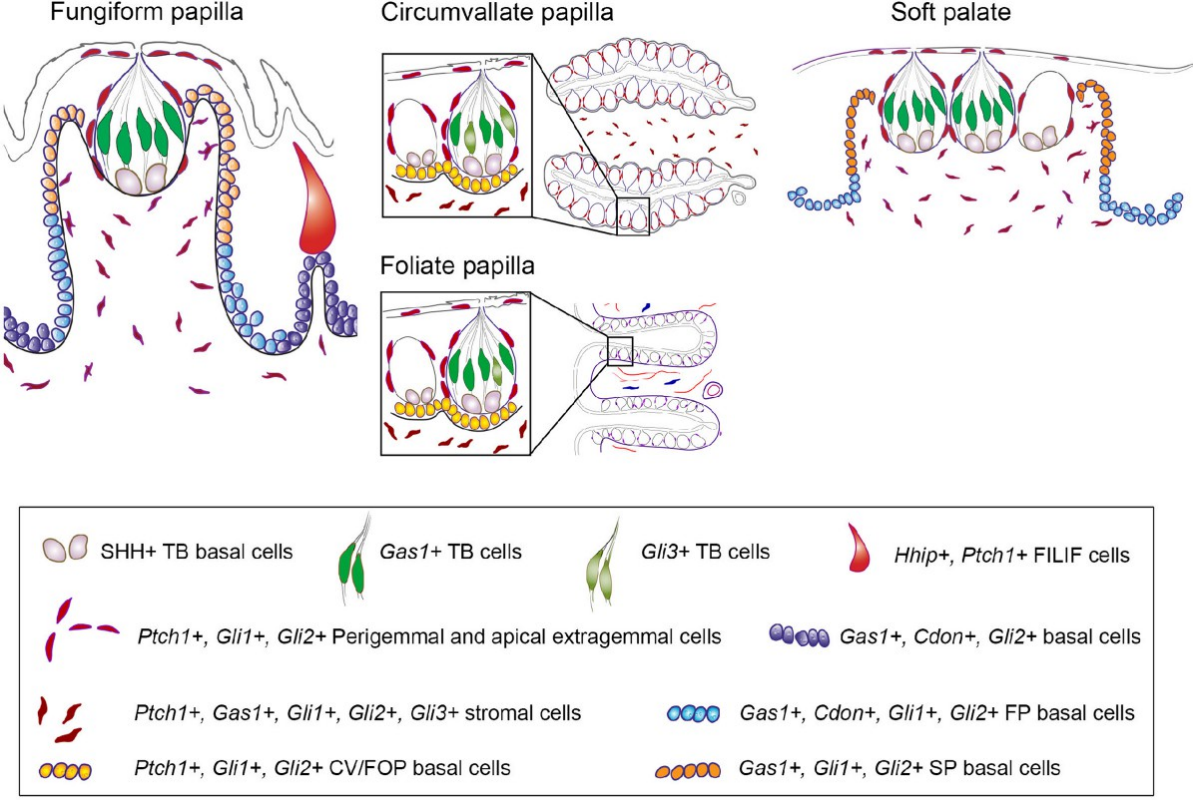
Expression patterns of HH signaling components in the adult FP, CVP, FOP and soft palate. Building from Fig 1A, HH signaling components are located in FP, CVP, FOP and soft palate. SHH is predominant in taste bud basal cells. *Gas1* is present in all papillae taste bud, whereas *Gli3* is also expressed in the posterior tongue papillae taste bud. *Hhip* and *Ptch1* are present in anterior epithelial face of FILIF. *Gli1*+, *Ptch1*+ and *Gli2*+ cells are observed in lingual basal, perigemmal, apical extragemmal and stromal cells of all the papillae. However, *Ptch1* is primarily present in the upper apical half of FP. On the other hand, HH co-receptors *Gas1* and *Cdon* are concentrated in lower half of FP wall and entire lingual epithelium. *Gas1* and *Gli3* are also expressed in the stromal cells of all the papillae and soft palate. The expression pattern of HH signaling components in the soft palate is similar to that observed in FP.

Building on our previous work exploring anterior-posterior tongue regulatory mechanisms [52], here we reveal similarities and differences in HH signaling activity within adult taste papillae (**Fig 9**). *Shh* ligand and the HH receptor *Gas1* exhibit similar expression patterns across all three papilla taste buds, suggesting a conserved homeostatic mechanism. On the other hand, *Gli3* is observed predominantly in posterior papilla taste buds (**Fig 9**). This contrasts with *Gas1* and *Cdon*, which are expressed in anterior tongue papilla epithelium (**Fig 9**), potentially contributing to the restriction of HH signaling to adult FP.

*Ptch1*, *Gli1* and *Gli2* are present in both anterior and posterior lingual basal, perigemmal and apical extragemmal cells (**Fig 9**). While *Ptch1*+ cells from FP basal cells become restricted to the apical half of FP (anterior tongue), apparently all basal cells in posterior tongue papillae retain *Ptch1* expression. A similar decline in *Ptch1* expression is also observed in the dental inner enamel epithelium to maintain SHH-responsive quiescent adult stem cells [76]. Our previous HH pathway inhibition studies showed a reduction in cell proliferation [25,26,28] and elimination of *Ptch1* [27], in apically lying basal cells, implying that *Ptch1* controls FP apical cell proliferation. While the basal half of FP retained cell proliferation, it could not prevent taste bud loss [25,26,28], which further suggests FP taste bud stem cell maintenance by *Ptch1*+ cells. The data highlight heterogeneous cell populations in the FP basal epithelium, which demarcate boundaries between the apical and basal epithelium (**Fig 9**). Further, the HH antagonist *Hhip* is specific to non-taste FILIF and is not observed in the taste papilla epithelium (**Fig 9**).

Stromal expression of HH receptors and GLI transcription factors is observed but whether they are co-expressed in the same subset of stromal cells remains to be investigated. We propose stromal diversity in the context of HH signaling components to create a distinct niche, as described previously [77].

### HH signaling in other oral tissue at adult stage

In addition to FP, taste buds are also housed in the soft palate (SP) epithelium without being enclosed in a papilla (**Fig 9**, soft palate). The maturation of SP taste buds is almost complete by P7 and is much faster than that of the taste buds of tongue papillae (after P21) [10]. Upon analyzing SP taste buds in adult mice, we found expression patterns similar to FP (**Fig 9**). *Shh* and *Gas1* are co-expressed in taste buds. The expression patterns of the HH receptors *Ptch1* and *Gas1*, and all three *Gli* transcription factors in mesenchyme corroborate a previous study conducted between embryonic days 13 and 14.5 [78]. In contrast to previous embryonic SP data [78], we observe epithelial expression of *Ptch1*, *Gas1*, *Gli1* and *Gli2* but not expression of *Hhip* in the adult SP. Whether these differences reflect a change in HH component expression or differences in technical approaches [78] remains to be investigated. Although *Cdon* was not studied previously, here we observed an expression pattern in the epithelium similar to *Gas1*.

We show that HH-responding *Gli1*+ cells are present in the SP epithelium, which give rise to taste buds [79]. When we treated adult rats with the HH pathway inhibition drug sonidegib, we observed that taste buds were reduced to half their normal numbers and the remaining taste buds were atypical [28] suggesting that HH signaling regulates adult SP taste buds. Embryonic SP epithelial-mesenchymal interactions, mediated by HH signaling, control palatal outgrowth [68,80–82]. Beyond embryonic HH mesenchymal signaling, in adult SP, our expression studies suggest paracrine signaling regulation from *Shh*+ taste buds and nerves to HH-responding palatal perigemmal, basal and stromal cells for tissue homeostasis.

### HH signaling in ganglia associated with gustatory system during adulthood

Multimodal chorda tympani, greater superficial petrosal and somatosensory lingual nerves receive afferents from the geniculate (GG) and trigeminal ganglion (TG) soma, respectively [83]. SHH is expressed in all GG and TG neurons [26,45]. Therefore, we see *Shh*+ nerves within the taste buds of FP that receive chorda tympani nerve fibers and SP that receive greater superficial petrosal nerve fibers. *Shh* expression in the lingual nerve innervating FP walls and FILIF connective tissue core is not clearly demonstrated even though TG neurons express SHH [28,46,47]. It is unclear whether this is a detection issue or reflects previously unappreciated neuronal heterogeneity.

Here, we reveal the expression patterns of other members of HH signaling (**Table 1**). Compared to the tongue or the soft palate, the GG and TG might utilize only positive regulators of HH signaling. While our findings indicate HH signaling likely regulates GG and TG, its precise impact on neuronal function and development needs further investigation.

In conclusion, our comparative analyses of unexplored HH pathway co-receptors and partially explored GLI transcription factors in anterior and posterior tongue, soft palate and ganglia, in epithelium, stroma or muscles reveals heterogeneity in their expression patterns. This work lays a foundation for understanding the intricate roles of HH signaling in distinct organs of the gustatory system. Future functional studies, guided by these expression maps, will dissect the regulatory mechanisms by which HH signaling components orchestrate tissue-specific development and function within the gustatory system.

## Supporting information

S1 Fig Expression pattern of HH signaling components in the anterior and posterior tongue and soft palate. (A, B) X-gal staining in *Ptch1*^*lacZ/+*^ reporter mouse indicates a reduction in *Ptch1*+ FP basal cells and taste bud cells at P19 (A) and expression in FILIF anterior epithelial face at P7 (B). (C-E) X-gal staining in *Gas1*^*lacZ/+*^ reporter mouse at P4, P21 and adult stages suggests that while lingual muscles express *Gas1*^*lacZ*^ at P4, *Gas1*+ muscle cells are not observed at P21 and adult tongues. Ecad antibody co-staining confirms absence of *Gas1*^*lacZ*^ expression in lingual epithelium at P4 (C). Epithelial *Gas1*^*lacZ*^ expression is observed at P21 (D) and maintained through the adult stage (E). (F,G) X-gal staining in *Cdon*^*lacZ/+*^ reporter mouse reveals stromal expression at P8 (F), which gets downregulated at adult stage (G). K8 antibody co-staining confirms no *Cdon* ^*lacZ*^ expression in taste bud (F). (H,I) X-gal staining in *Hhip*^*lacZ/+*^ reporter mouse shows stromal *Hhip*^*lacZ*^ expression at P12 in tongue (H) and below the taste bud (H, inset, arrow) but not at P30 (I). (J) X-gal staining in P14 *Gli1*^*lacZ/+*^ reporter mouse indicates reduced *lacZ* expression in FILIF (arrow). (K, L) X-gal staining in *Shh*^*lacZ/+*^ reporter mouse shows expression in the taste buds of CVP at P7 (K) and soft palate at adult stage (L). Black dotted lines outline the epithelium. Scale bars are 50μm.
(TIF)
