# Supplementary figures and images for: Distinct expression patterns of Hedgehog signaling components in mouse gustatory system during postnatal tongue development and adult homeostasis
Source: PLoS One. 2024 Jun 7;19(6):e0294835. doi: 10.1371/journal.pone.0294835 (PMC11161123; doi:10.1371/journal.pone.0294835)

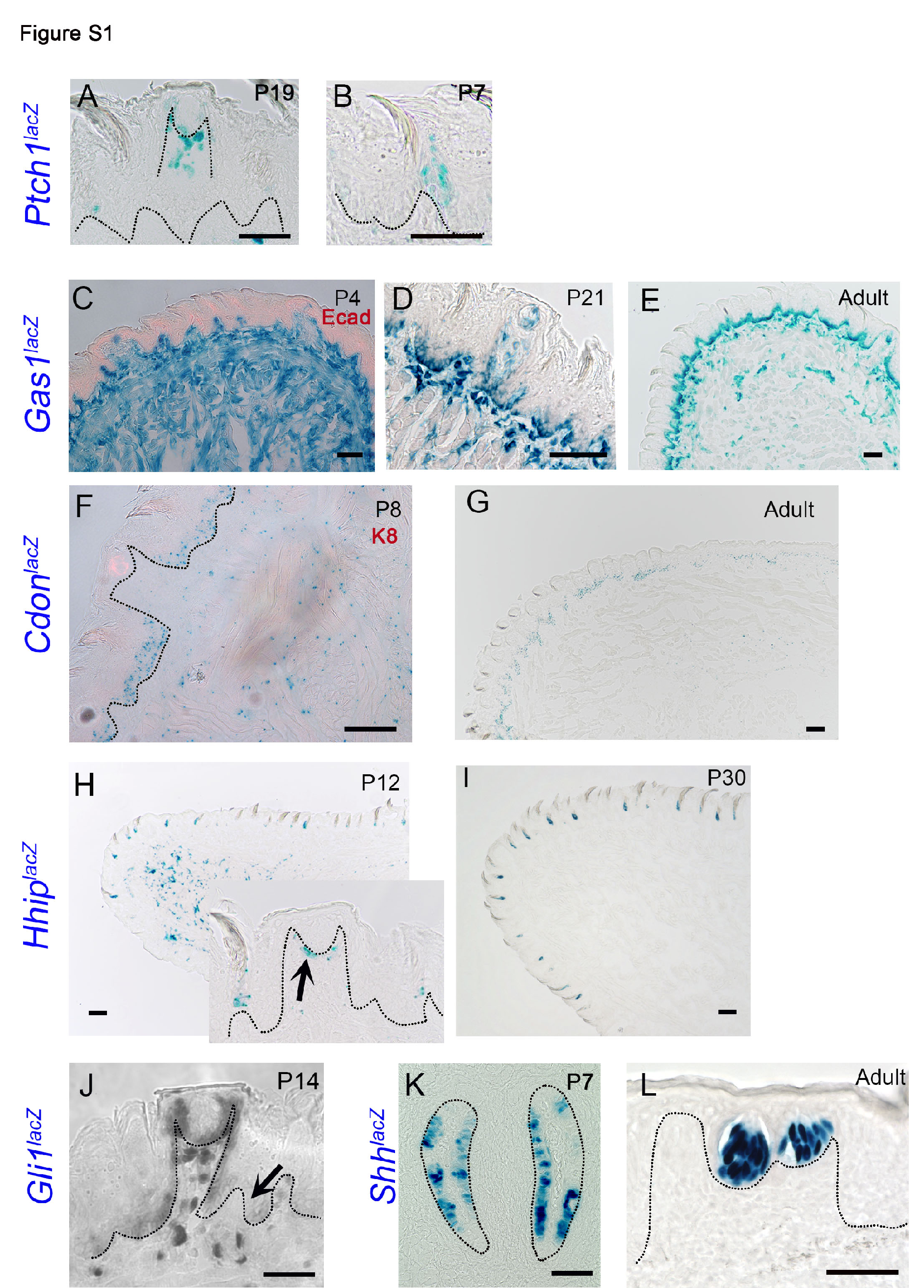

Supplement: S1 Fig — (A, B) X-gal staining in Ptch1lacZ/+ reporter mouse indicates a reduction in Ptch1+ FP basal cells and taste bud cells at P19 (A) and expression in FILIF anterior epithelial face at P7 (B). (C-E) X-gal staining in Gas1lacZ/+ reporter mouse at P4, P21 and adult stages suggests that while lingual muscles express Gas1lacZ at P4, Gas1+ muscle cells are not observed at P21 and adult tongues. Ecad antibody co-staining confirms absence of Gas1lacZ expression in lingual epithelium at P4 (C). Epithelial Gas1lacZ expression is observed at P21 (D) and maintained through the adult stage (E). (F,G) X-gal staining in CdonlacZ/+ reporter mouse reveals stromal expression at P8 (F), which gets downregulated at adult stage (G). K8 antibody co-staining confirms no Cdon lacZ expression in taste bud (F). (H,I) X-gal staining in HhiplacZ/+ reporter mouse shows stromal HhiplacZ expression at P12 in tongue (H) and below the taste bud (H, inset, arrow) but not at P30 (I). (J) X-gal staining in P14 Gli1lacZ/+ reporter mouse indicates reduced lacZ expression in FILIF (arrow). (K, L) X-gal staining in ShhlacZ/+ reporter mouse shows expression in the taste buds of CVP at P7 (K) and soft palate at adult stage (L). Black dotted lines outline the epithelium. Scale bars are 50μm. (TIF) [file pone.0294835.s001.tif]
